# Modified wound dressing with phyto-nanostructured coating to prevent staphylococcal and pseudomonal biofilm development

**DOI:** 10.1186/1556-276X-7-690

**Published:** 2012-12-31

**Authors:** Ion Anghel, Alina Maria Holban, Alexandru Mihai Grumezescu, Ecaterina Andronescu, Anton Ficai, Alina Georgiana Anghel, Maria Maganu, Veronica Lazǎr, Mariana Carmen Chifiriuc

**Affiliations:** 1 ENT (Otolaryngology) Department, Coltea Hospital, Carol Davila University of Medicine and Pharmacy, IC Bratianu No. 1, Bucharest, 030171, Romania; 2 Department of Microbiology, Faculty of Biology, Universtity of Bucharest, Aleea Portocalelor No. 1-3, Bucharest, 060101, Romania; 3 Department of Science and Engineering of Oxidic Materials and Nanomaterials, Faculty of Applied Chemistry and Materials Science, University Politehnica of Bucharest, Polizu Street No. 1-7, Bucharest, 011061, Romania; 4 ENT (Otolaryngology) Department, Coltea Hospital, Carol Davila University of Medicine and Pharmacy, IC Bratianu no 1, 030171, Bucharest, Romania; 5 Center of Organic Chemistry “Costin D. Nenitescu”, Romanian Academy, 202B Splaiul Independentei, Bucharest, 050461, Romania; 6 Doctor Anghel Medical Center, Theodor Sperantia Street, Bucharest, 30932, Romania

**Keywords:** Magnetite nanoparticles, Eugenol, Limonene, Wound dressing

## Abstract

This paper reports a newly fabricated nanophyto-modified wound dressing with microbicidal and anti-adherence properties. Nanofluid-based magnetite doped with eugenol or limonene was used to fabricate modified wound dressings. Nanostructure coated materials were characterized by TEM, XRD, and FT-IR. For the quantitative measurement of biofilm-embedded microbial cells, a culture-based method for viable cell count was used. The optimized textile dressing samples proved to be more resistant to staphylococcal and pseudomonal colonization and biofilm formation compared to the uncoated controls. The functionalized surfaces for wound dressing seems to be a very useful tool for the prevention of wound microbial contamination on viable tissues.

## Background

Humans are natural hosts for many bacterial species that colonize the skin and mucosa as normal microbiota. However, in certain conditions, some microbes composing our microbiota generically called opportunistic pathogens can cause serious infections mainly by regulating their virulence [1,2]. Predisposing factors to cutaneous infections include minor trauma, pre-existing skin disease, poor hygiene, and, rarely, impaired host immunity [3]. Based on World Health Organization report in 2011, skin diseases still remain common in many rural communities in developing countries, with serious economic and social consequences, as well as health implications.

As a form of adaptability and evolution, bacteria managed to establish a well-organized behavior into a very efficient assembly, called biofilm. Bacterial biofilm formation is the prevailing microbial lifestyle in natural and man-made environments and occurs on all surface types, including biological surfaces; it can be defined as a community of microorganisms irreversibly attached to a surface, producing extracellular polymeric substances, exhibiting an altered phenotype compared with corresponding planktonic cells, and interacting with each other [4,5]. One of the most significant clinical aspects is the fact that bacterial biofilms cause chronic infections because they disclose increased tolerance to antibiotics and disinfectants, as well as resisting phagocytosis and other components of the body’s defense system [6]. Approximately, 80% of all human infections are associated with biofilms, and evidence for their role in an ever-growing number of cutaneous disorders is constantly unfolding [7].

In the recent years, researchers aimed to find alternative methods of dealing with infections with biofilm-embedded bacteria, knowing that adherent microbial cells exhibit high antibiotic resistance. One of the most efficient strategies is to interfere with bacterial adherence, the first step in the biofilm formation, by direct blockage of surface receptors [8] or using a non-specific strategy, usually involving compounds with anti-adherence properties [9-11]. Another efficient strategy seems to be the one involving the manipulation of communication processes between bacteria into the biofilm, using different natural or artificially synthetized compounds [12-14]. Bearing in mind that chemically synthetized compounds may be toxic and have usually unpredictable long-term effect on the mammalian host cell, natural compounds exhibiting anti-microbial activity are considered as a more preferred alternative [15,16]. Essential vegetal oils are natural compounds that have proved to be highly efficient as antimicrobial agents, demonstrating significant anti-adherence and anti-biofilm properties [17,18]. However, the use of essential oils can be limited by their high volatility and low stability [19].

Magnetic iron oxide nanoparticles have appeared as a well-established technology and an important research field, mainly because of their superparamagnetism properties that allow to be guided with an external magnetic field, [20,21]. Potential applications in the field of biotechnology and nanomedicine such as biomagnetic separations [22], biosensors [23], carriers for targeted drug delivery [24-28], hyperthermia-producing systems [29], inhibition of biofilm development [30,31], stabilization of essential oils [32], and contrast agents in magnetic resonance imaging [33,34] have been proposed. The material surface chemistry and the electronic configuration of the surface complexes have major influences on the reactivity and properties [35].

In this paper, we report preliminary data on new magnetite-based nanostructures used to create nanofluids with both microbicidal and anti-adherence properties, and to evaluate their potential to improve the anti-biofilm properties of a cotton-based material, routinely used for covering cutaneous wounds. The anti-adherence and anti-biofilm properties of this nano-modified wound dressing were assessed *in vitro* using two strains belonging to bacterial species commonly found in wound infections, i.e., *Pseudomonas aeruginosa* and *Staphylococcus aureus*.

## Methods

### Materials

All chemicals were used as received. FeCl_3_, FeSO_4_ · 7H_2_O, NH_3_, sodium palmitate (C_16_), CHCl_3_, and CH_3_OH were purchased from Sigma-Aldrich Chemie GmbH (Munich, Germany). The textile wound dressing represented by 1 × 1-cm sections were obtained from the Otolaryngology Department of Coltea Hospital, Bucharest, Romania.

### Fabrication of nanostructure

Magnetic iron oxide particles are usually prepared by wet chemical precipitation [36,37] from aqueous iron salt solutions by means of alkaline media, like NH_3_. Half gram of sodium palmitate (C_16_) was solubilized in a known volume of ultrapure water, corresponding to a 1.00% (w/w) solution, under stirring at room temperature. Then, 4 mL of a basic aqueous solution consisting of 28% NH_3_ was added to C_16_ dispersion. Thereafter, 100 mL of FeSO_4_/FeCl_3_ (molar ratio 2:1) was dropped under permanent stirring up to pH = 8 [38,39]. The product (Fe3O4@C_16_) was repeatedly washed with methanol, separated with a strong NdFeB permanent magnet, and subsequently dried in an oven at 40°C, until reaching a constant weight.

### Characterization of nanostructure

#### FT-IR

A Nicolet 6700 Fourier transform infrared spectroscopy (FT-IR) spectrometer (Thermo Nicolet, Madison, WI, USA) connected to the software of the OMNIC operating system (version 7.0 Thermo Nicolet) was used to obtain FT-IR spectra of hybrid materials. The samples were placed in contact with attenuated total reflectance on a multibounce plate of ZnSe crystal at controlled ambient temperature (25°C). FT-IR spectra were collected in the frequency range of 4,000 to 650 cm^−1^ by co-adding 32 scans and at a resolution of 4 cm^−1^ with strong apodization. All spectra were ratioed against a background of an air spectrum.

#### XRD

X-ray diffraction analysis (XRD) was performed on a Shimadzu XRD 6000 diffractometer (Shimadzu Corporation, Kyoto, Japan) at room temperature. In all the cases, CuKα radiation from a Cu X-ray tube (run at 15 mA and 30 kV) was used. The samples were scanned in the Bragg angle 2*θ* range of 10 to 80.

#### TEM

The transmission electron microscopy (TEM) images were obtained on finely powdered samples using a Tecnai™ G2 F30 S-TWIN high resolution transmission electron microscope from FEI Company (OR, USA) equipped with EDS and SAED. The microscope was operated in transmission mode at 300 kV with TEM point resolution of 2 Å and line resolution of 1 Å. The fine MNP powder was dispersed into pure ethanol and ultrasonicated for 15 min. After that, diluted sample was put onto a holey carbon-coated copper grid and left to dry before TEM analysis.

#### DTA-TG

The thermogravimetric (TG) analysis of the biocomposite was assessed with a Shimadzu DTG-TA-50H instrument. Samples were screened to 200 mesh prior to analysis, were placed in alumina crucible, and heated with 10 K · min^−1^ from room temperature to 800°C, under the flow of 20 mL · min^−1^ dried synthetic air (80% N_2_ and 20% O_2_).

### Fabrication of the hybrid phyto-nanostructure

Magnetic nanostructure Fe_3_O_4_@C_16_ (200 mg) was solubilized in 1 mL of chloroform and oriented in magnetic field, and 100 μL analytical standard of eugenol (E) (Sigma-Aldrich) and respectively, limonene (L) (Sigma-Aldrich) were added and mixed until complete evaporation of chloroform was reached. This step was repeated three times for the uniform loading of E and L in the core-shell nanostructure.

### Fabrication of the modified wound dressing coated with the phyto-nanostructure

**Figure 1:**
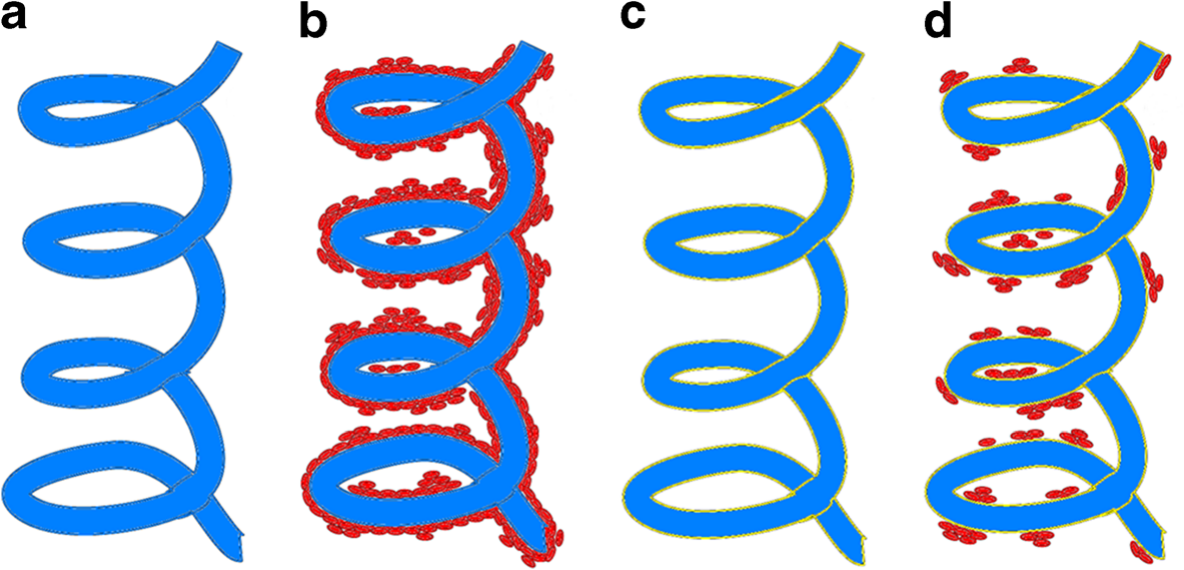
**Schematic representation of the microbial biofilm development on the uncoated and coated wound dressings.** (**a**) wound dressing fiber; (**b**) biofilm development on the surface of wound dressing fiber; (**c**) coated wound dressing fiber by the obtained phyto-nanofluid; (**d**) poorly developed microbial biofilm on the surface of the modified textile material.

The layer of phyto-nanostructure on the wound dressing material was achieved by submerging the wound dressing pieces (1 × 1 cm) in 5 mL of phyto-nanofluid (Fe_3_O_4_@C_16_/E or Fe_3_O_4_@C_16_/L:chloroform = 1 mg/mL), and the wound dressing pieces have been extemporaneously dried at room temperature. The rapid drying was facilitated by the convenient volatility of chloroform [40]. The phyto-E and phyto-L-nanomodified wound dressing specimens were sterilized by ultraviolet irradiation for 20 min. Figure 1 illustrates the wound dressing with phyto-nanofluid coating.

### Bacterial adherence and biofilm assay by viable cell count method

Overnight bacterial cultures of *P. aeruginosa* ATCC 27853 and *S. aureus* ATCC 25923 were diluted in fresh Luria broth (LB) up to a turbidity of 0.5 McFarland (approximately 1 × 10^8^ CFU/mL), and 2 mL of the obtained suspension were seeded in 6 multi-well plates containing the wound dressing specimens previously sterilized by UV irradiation. The plates were incubated for 24 h at 37°C. For the adherence assay, after the incubation time, the materials were gently washed with sterile phosphate buffered saline (PBS) in order to remove the non-adherent bacteria and placed in 2 mL centrifuge tubes containing 1 ml of sterile PBS. The samples were vigorously mixed by vortexing for 1 min and sonicated for 10 s [41]. Serial dilutions obtained from each sample were inoculated on LB agar plates in triplicates, and viable cell counts (VCCs) were assessed after incubation for 24 h at 37°C. For the biofilm assay, the materials containing attached bacteria were washed with sterile PBS and incubated in fresh LB broth for 24 h, 48 h, and 72 h at 37°C. After each incubation period, the samples were gently washed with sterile PBS, mixed by vortexing, and sonicated. Serial dilutions were placed on LB plates in triplicate. After 24 h of incubation at 37°C, VCCs were assessed. The experiment was repeated with three separate occasions.

### Statistics

For the statistical interpretation, we have used GraphPadInStat (GraphPad Software, Inc., CA, USA) and Prism softwares (Prism Software Corporation, CA, USA). The results were analyzed and compared using one-way analysis of variance (ANOVA) and Bonferroni Multiple Comparisons Test. *P* values lower than 0.05 were considered significant.

## Results and discussion

Textile industry is a small part of the global research in the emerging areas of nanotechnology, the fibers and textiles industries being in fact the first to have successfully implemented these advances and demonstrated the applications of nanotechnology for consumer usage [42]. Nanotechnologies have been largely used for different biomedical applications.

In our previous papers, we have demonstrated by scanning electron microscopy the ability of Fe_3_O_4_@C_18_ to prevent the fungal adherence of *Candida albicans* on optimized textile dressing samples coated with functionalized magnetite nanoparticles, as compared to uncoated materials [36]. These functionalized Fe_3_O_4_@C_18_ nanoparticles exhibited also the ability to stabilize, limit the volatilization, and potentiate the fungicidal effect of *Salvia officinalis* essential oil [43]. On the other hand, limonene and eugenol, the major compounds of essential oils extracted from *Anethum graveolen*s (56.53%) and *Eugenia caryophyllata* (92.45%) proved, to exhibit very good antimicrobial properties [28,44]. In this paper, we report the successful fabrication of two phyto-nanofluids for coating textile wound dressings, based on limonene and eugenol loaded in magnetic nanoparticles, in order to increase their microbicidal and anti-biofilm properties and, thus, combat the cutaneous opportunistic infections.

**Figure 2:**
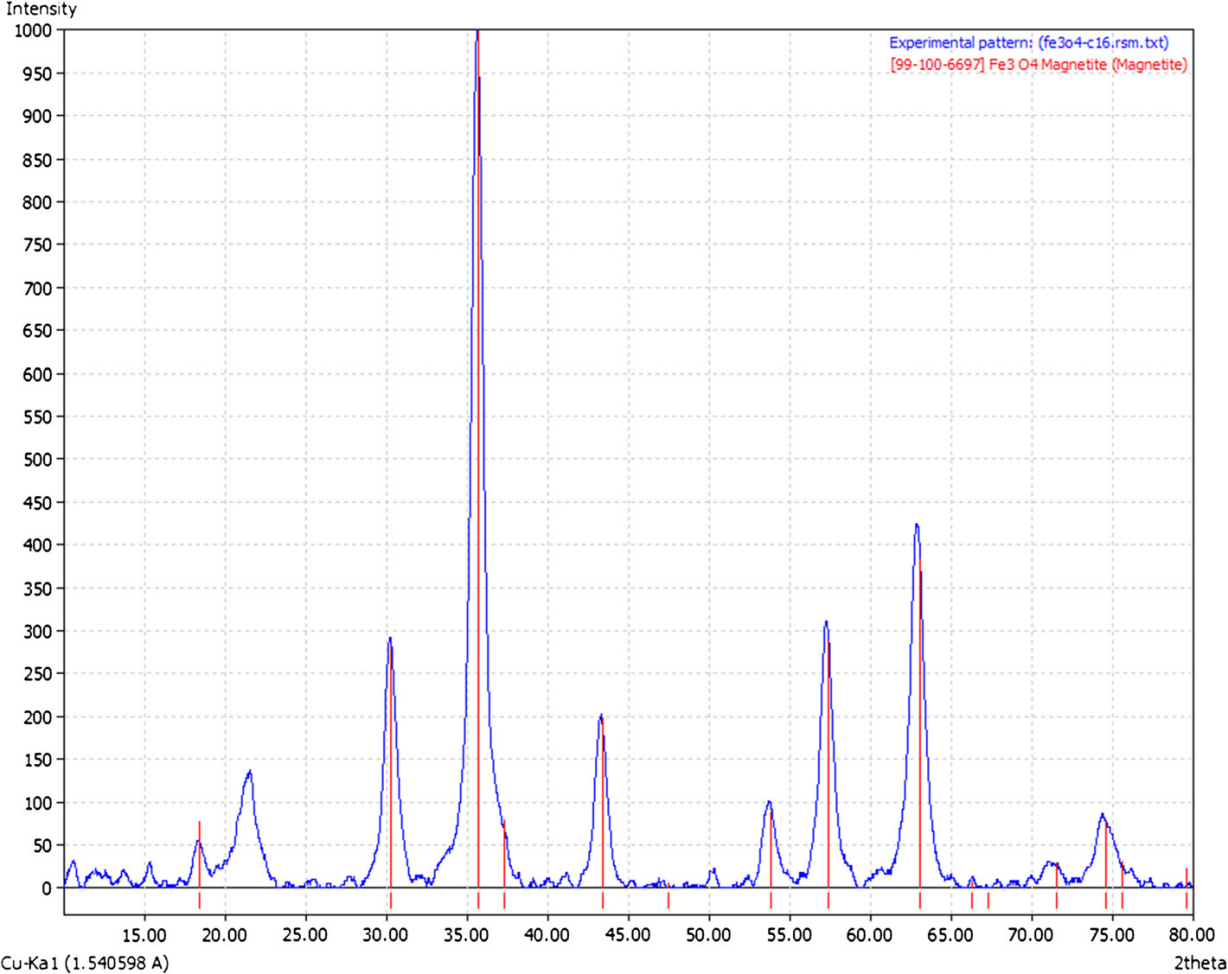
XRD pattern of the nanostructure.

**Figure 3:**
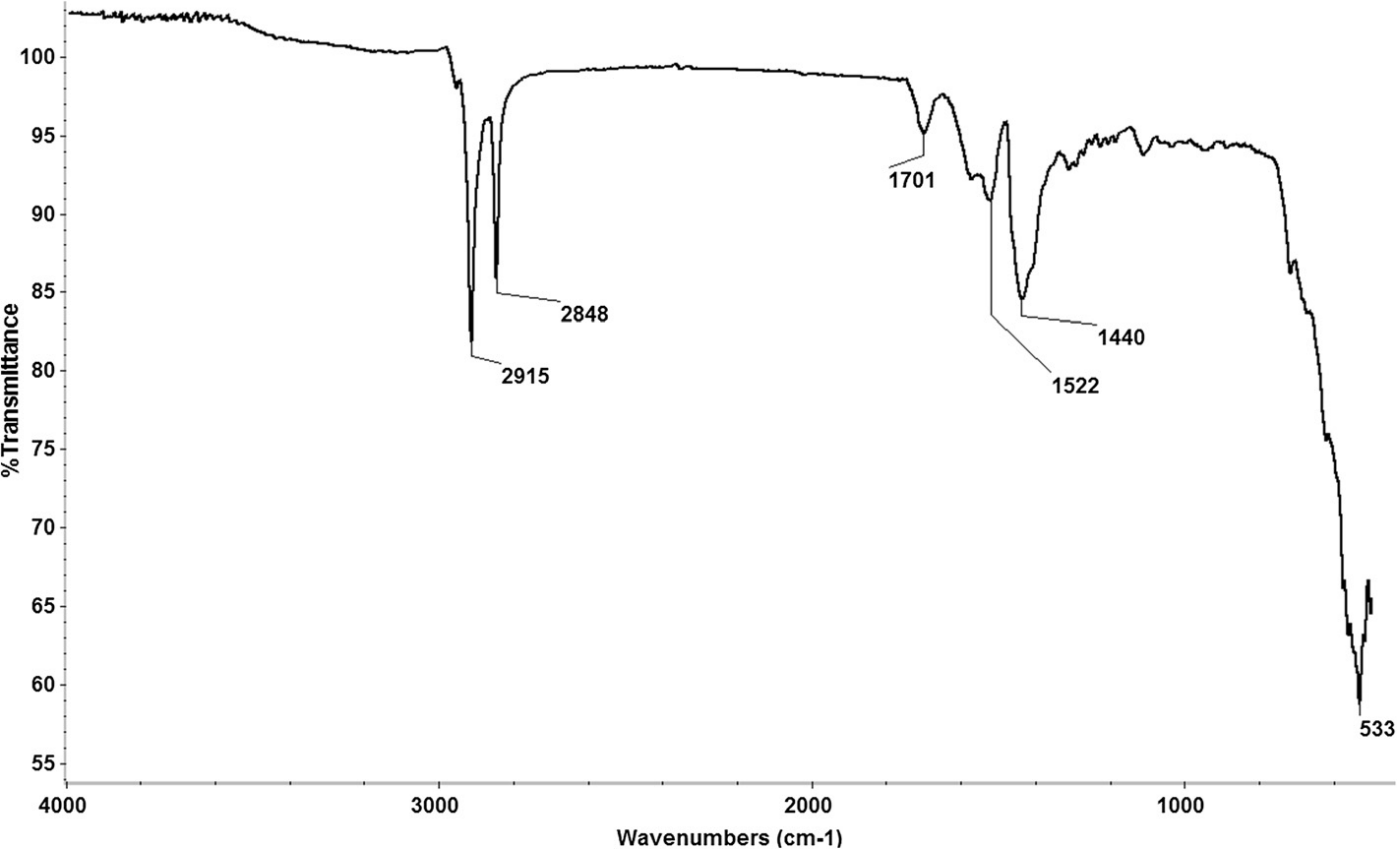
FT-IR spectrum of the nanostructure.

**Figure 4:**
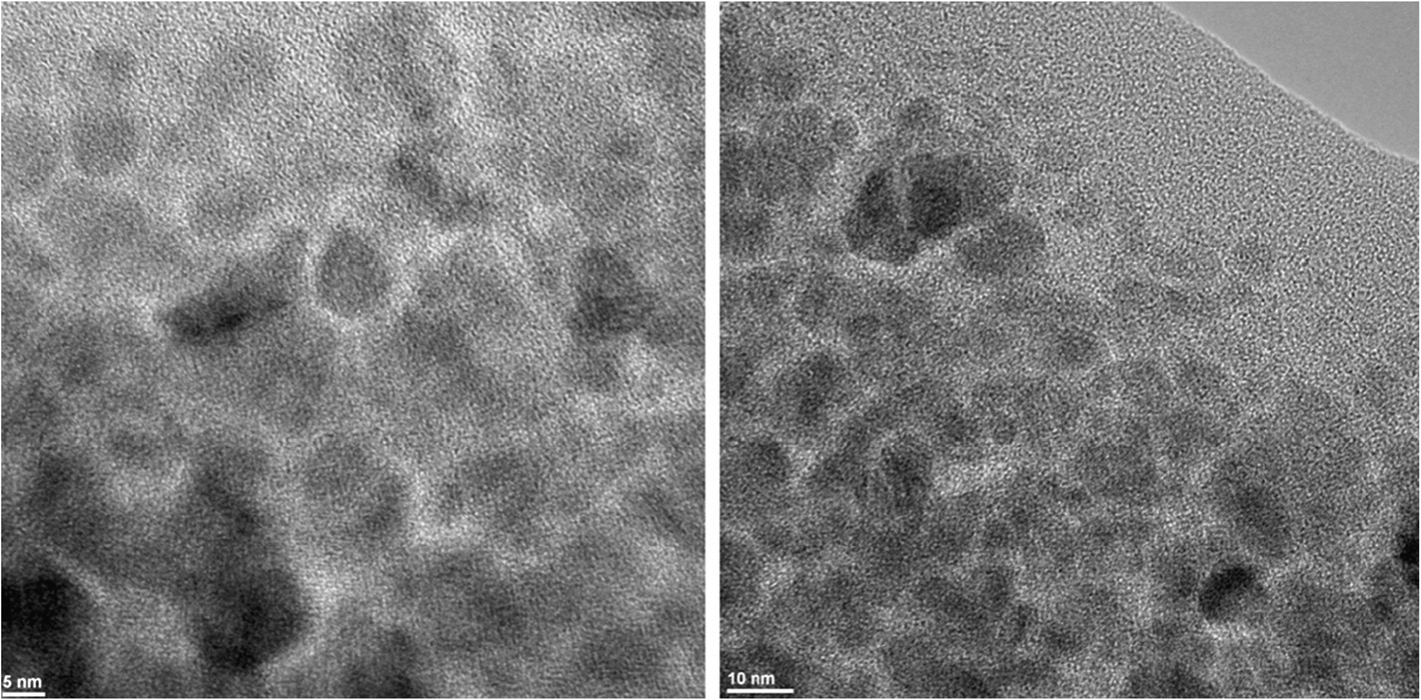
HR-TEM images of the fabricated nanostructure.

**Figure 5:**
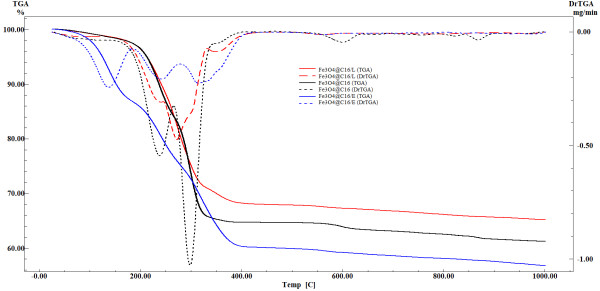
TGA diagram of fabricated nanostructures.

The obtained nanostructure was characterized by XRD as illustrated in Figure 2, and the results showed that the diffraction patterns and the relative intensities of all diffraction peaks match well with magnetite (based on ICDD 82–1533). Also, the sample has the characteristics of bulk magnetite crystallite phase, and the broad peaks suggest the nanocrystallite nature of magnetite particles [45,46], the average crystallite size being 10.58 nm (based on Scherrer formula). FT-IR spectrum of the nanostructure exhibits a characteristic broad peak of magnetite at about 533 cm^−1^ (Fe-O stretching) [47]. The FT-IR analysis also identified the organic coating on the surface of the magnetite nanoparticles (Figure 3). The peaks recorded at about 1,572 and 1,701 cm^−1^ at FT-IR spectrum of the nanostructure can be assigned to structures of the type COO^−^M^+^. The peaks at 2,915 and 2,848 cm^−1^ were assigned to stretching vibration of C-H (Figure 3). The nanostructure diameter was approximated from the TEM images (as presented in Figure 4), showing that the particles are spherical with an average size of 10 nm which, corroborated with the XRD data, means that the obtained nanoparticles are formed by only one crystallite. The presence of essential oils induces a strong modification of the thermal behavior of the two nanostructured materials (Figure 5). In the case of phyto-E-nanostructurated material, the weight loss increases with about 4.6%, which can be mainly attributed to the eugenol adsorption onto the nanomaterial. The weight loss was surprisingly affected in the phyto-L-nanostructurated material, where the weight loss became even lower than that corresponding to Fe_3_O_4_@C_16_. We explain this anomaly by the fact that limonene and C_16_ interact by special hydrophobic interactions, and the complex may be partially lost during the drying step.

Due to their widespread, easy manipulation, and low side effects, direct contact wound absorptive natural-based plasters are preferred for wound dressing. Specialized literature reports few studies aimed to improve the quality and antibacterial properties of natural or artificial materials used for wound dressing and covering, but the proposed techniques are mainly based on using artificial, new chemically synthetized compounds [16,17].

Essential oils represent an alternative for treating microbial infections because they are natural vegetal compounds with lower or no side effects for the host compared with artificially synthetized antimicrobial compounds, representing one of the ecological anti-infectious strategies. However, their effects can be impaired by their great volatility, highlighting the necessity of novel vectoring stabilizing systems. In the recent years, the usage of nanosystems for clinical issues has emerged, mainly because of their reduced structures and their proved characteristics, as antimicrobial activity. Even though nanosystems are considered a novel challenge for medicine, their usage is largely restricted because of their unknown long term effects and sometimes because of their toxicity on eukaryotic cells. During this study, we have investigated the possibility of improving the antimicrobial activity of wound dressings by modifying their surface using a nanofluid to assure the stability and controlled release of some volatile organic compounds isolated from essential oils. Our results obtained on two *in vitro* monospecific bacterial biofilm models involving cotton-based wound dressers layered with a phyto-nanostructured coating demonstrated that the functionalized textile materials exhibited antimicrobial effects on wound-related pathogens.

**Figure 6:**
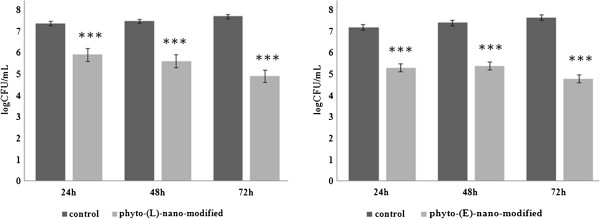
**The logarithmic values VCCs of *S. aureus *cells adhered and embedded in biofilms formed on the wound dressing surface: uncoated *vs. *phyto-L and E-nano-modified.** Triple asterisk denotes *P* < 0.001; indicated samples *vs.* uncoated control based on one way ANOVA test.

**Figure 7:**
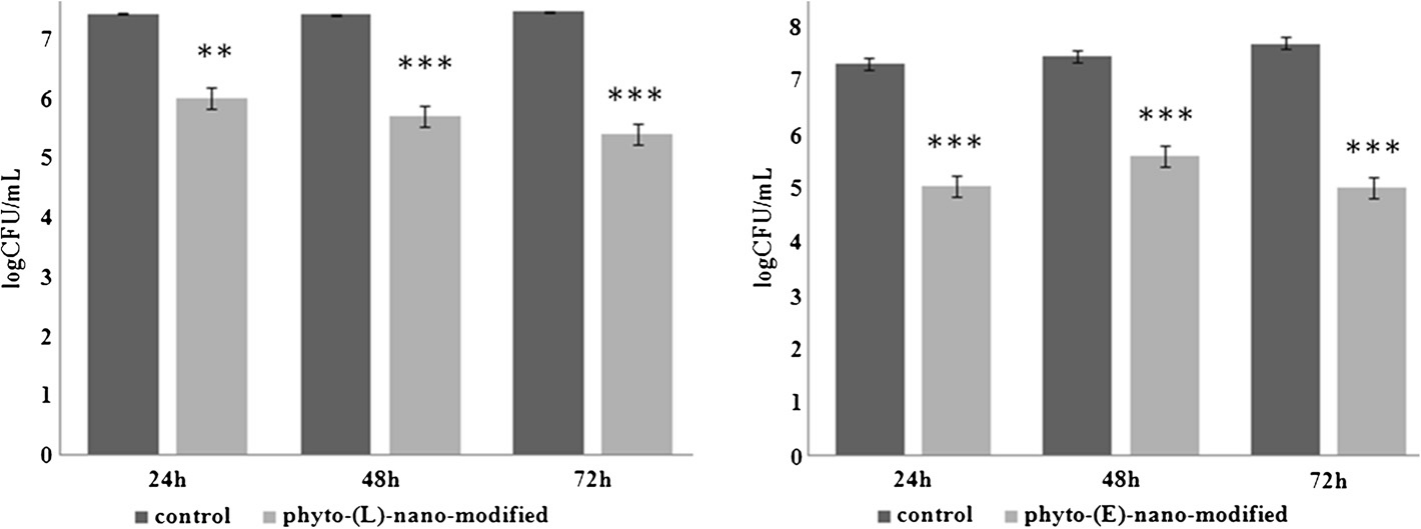
**The logarithmic values of viable cell counts of *P. aeruginosa *cells.** The cells adhered and embedded in biofilms and formed on the wound dressing surface: uncoated vs. nanophyto-L and E-modified. Double asterisk denotes *P* < 0.01; triple asterisk, *P* < 0.001. Indicated samples *vs.* uncoated control based on one way ANOVA test.

VCCs assessed from mechanically detached biofilm bacteria revealed a slightly different ability of the two modified wound dressings. The results revealed that the nanofluid coating containing L affected both the initial stage of biofilm formation and the development of a mature biofilm, as demonstrated by the lower VCCs obtained at the three harvesting time intervals (i.e., 24 h, 48 h, and 72 h), as comparing with control, uncoated textile materials (*P* < 0.0001). Even though *P. aeruginosa* ATCC 27853 grew better, the differences between *S. aureus* and *P. aeruginosa* VCC values were not significantly different. The nanofluid exhibiting comparative antibiofilm effects in both models (Figure 5) induced a significantly reduced biofilm development expressed as viable cells in time (*P* < 0.05). The phyto-E-nano-modified wound dressing model has proved to have also a significant antibiofilm activity, determining a pronounced biofilm inhibition on both *S. aureus* (Figure 6) and *P. aeruginosa* (Figure 7) models at all three tested time points (*P* < 0.0001). The effect of this system seems to be more pronounced on adherence and initial biofilm formation compared to the L-based one, in case of *P. aeruginosa*.

For both tested phyto-nanosystems, the most important decrease of VCCs was observed at 72 h, demonstrating the ability of the obtained nanostructure to reduce the volatility of the essential oils and to assure their release in active forms for the entire duration of the experiment. Taken together, our data demonstrate that the obtained phyto-nanofluids are very useful for the stabilization and controlled release of some antimicrobial active compounds, such as the essential oil major compounds with antimicrobial activity, eugenol and limonene. The fabricated nanostructures with an adsorbed shell of L and E compounds are much more efficient in triggering bacterial biofilm disruptions.

## Conclusions

In this paper, we report a successful antimicrobial system represented by modified wound dressing coated by a hybrid nanofluid based on magnetite and natural compounds of vegetal origin, i.e., eugenol and limonene, with a great potential of application in wound healing. The functionalized textile material cumulate the anti-adherent properties of magnetite and microbicidal activity of eugenol and limonene, exhibiting significant anti-adherence and anti-biofilm properties against two of the bacterial pathogens most frequently implicated in the etiology of cutaneous wound infections. The tested nanofluid proved to be efficient for stabilizing and controlling the release of volatile natural compounds, thus maximizing their biological activity. The proposed phyto-nanostructures are recommended to be used as a fixed layer on a regular external wound cover. Their topical application at cutaneous level minimizes the risk of toxicity effects normally associated with an implanted device.

## Competing interests

The authors declare that they have no competing interests.

## Authors’ contributions

AMH and IA conceived the study, provided the microbial strains, and drafted the manuscript together with AMG and MCC. AMG, AF and MM performed the synthesis and characterization of nanofluid. AMG obtained the essential oil. AMH and AGA performed the biological analyses. EA and VL participated in the design of the study and coordination. All authors read and approved the final manuscript.
